# LAPTM4B polymorphism increases susceptibility to multiple cancers in Chinese populations: a meta-analysis

**DOI:** 10.1186/1471-2156-15-48

**Published:** 2014-04-18

**Authors:** Cuiju Mo, Yu Lu, Yan Deng, Jian Wang, Li Xie, Taijie Li, Yu He, Qiliu Peng, Xue Qin, Shan Li

**Affiliations:** 1 Department of Clinical Laboratory, First Affiliated Hospital of Guangxi Medical University, Nanning, Guangxi 530021, China

**Keywords:** *LAPTM4B*, Polymorphism, Cancer, Meta-analysis

## Abstract

**Background:**

Lysosome-associated protein transmembrane-4 beta (*LAPTM4B*) is a novel cancer-related gene. While recent studies have reported that the *LAPTM4B* polymorphism increased the susceptibility of several cancers, the results remain inconclusive. Therefore, we performed a meta-analysis to systematically summarize the possible association.

**Results:**

The meta-analysis was conducted based on 17 studies in Chinese populations, including 4160 cases and 4148 controls. The relevant studies were searched through electronic databases updated in November 2013. The strength of association between the *LAPTM4B* polymorphism and susceptibility to multiple cancers was assessed by odds ratio (OR) and 95% confidence interval (95% CI).

The meta-analysis results suggested that the *LAPTM4B* polymorphism was significantly associated with overall susceptibility to multiple cancers in all genetic models (*2 vs. *1, OR = 1.53, 95% CI = 1.37–1.70; *2/2 vs. *1/1, OR = 2.18, 95% CI = 1.72–2.75; *2/1 vs.*1/1, OR = 1.62, 95% CI = 1.41–1.86; *2/1 + *2/2 vs. *1/1, OR = 1.70, 95% CI = 1.47–1.97; *2/2 vs. *2/1 + *1/1, OR = 1.76, 95% CI = 1.50–2.05). Further subgroup analysis revealed a significant association between the *LAPTM4B* polymorphism and cancer susceptibility in the subgroups stratified by control source, cancer type, histopathologic differentiation, and TNM stage.

**Conclusions:**

This meta-analysis indicated that the *LAPTM4B* *2 allele was associated with increasing risk of multiple cancers, tumor initiation and development.

## Background

Cancer is one of the leading causes of death in both developed and developing countries, genetic factors contribute significantly to carcinogenesis [1]. Lysosome-associated protein transmembrane-4 beta (*LAPTM4B*) is a novel cancer-related gene that was cloned from hepatocellular carcinoma (HCC) tissues using fluorescence differential display, rapid amplification of cDNA ends (RACE), and reverse transcription polymerase chain reaction (RT–PCR) [2,3]. According to BLAST program analysis, the *LAPTM4B* gene is composed of seven exons and six introns mapped to chromosome 8q22 [4]. The full length of messenger RNA for this gene is 2245 bp, which encoded a lysosome-associated protein with four transmembrane regions. Because it is 46% homologous with a human lysosome-associated transmembrane-4 protein (LAPTM4A) at the amino acid level, it was named *LAPTM4B* by the International Committee [5].

In previous studies, *LAPTM4B* mRNA and protein were found to be overexpressed in HCC tissues and expressed at fairly low levels in noncancerous or normal liver tissues. In addition, the expression levels were associated with the differentiation status of HCC; it exhibited higher expression in poor-differentiated HCC than in moderate and well-differentiated HCC [4-6]. Recently, upregulated *LAPTM4B* expression was also found in many other malignant tumors, such as breast cancer [7], pancreatic cancer [8], and gallbladder carcinoma (GBC) [9]. *LAPTM4B* has important roles in many biological processes, including malignant transformation; cell proliferation, invasion, metastasis, and apoptosis; and signal transduction [9,10].

*LAPTM4B* has two alleles: *LAPTM4B* *1 (GenBank accession number AY219176) and *LAPTM4B* *2 (GenBank accession number AY219177) [11]. The difference between allele *1 and allele *2 is at the 5’UTR in the first exon; allele *1 contains only a single 19-bp segment, whereas allele *2 has two tandem repeats of the 19-bp segment. *LAPTM4B* allele *1 produces a 317-amino acid protein, while allele *2 was predicted to encode a 370-amino acid protein; there are 53 extra amino acids at the N-terminus of the proteins produced by allele *2 [4]. Recent studies have reported that the *LAPTM4B* polymorphism is associated with cancer risk. The *LAPTM4B* *2 allele is reportedly associated with increased risk of such cancers as liver, gastric, colon, and gallbladder cancer, but not with rectal, oesophageal, or pancreatic carcinoma. Given these controversial results and the limits of a single study with a small sample size, we performed the present meta-analysis of all eligible studies in Chinese populations to investigate the association between the *LAPTM4B* polymorphism and susceptibility to multiple cancers.

## Methods

### Search strategy

The relevant studies were searched through the PubMed, Embase, Google Scholar, Wanfang, and China National Knowledge Infrastructure (CNKI) electronic databases updated in November 2013. There were no languages, time period, or sample size restrictions. The search key words were “cancer, carcinoma, tumor, neoplasms”, “*LAPTM4B*, Lysosome-associated protein transmembrane-4”, and “polymorphism, mutation, variant”. Reference lists of retrieved articles and reviews were checked for other eligible studies. If the same case series was researched by more than one study, only the one with the largest population was included in our study. If one study investigated multiple cancers, each cancer type was counted as a separate comparison in the subgroup stratified by cancer type.

### Inclusion and exclusion criteria

Studies included in the meta-analysis had to meet the following inclusion criteria: (1) original case-control studies; (2) associated the *LAPTM4B* polymorphism with cancer susceptibility in a Chinese population; (3) provided sufficient information of genotype distribution in the cases and controls; (4) did not repeat reports in the same population. The criteria for exclusion were (1) case reports, reviews, overlapped data, animal or mechanism studies; (2) no genotype frequency or genotype information provided; (3) not enough information for data extraction.

### Data extraction

The following data were independently extracted from all eligible studies by two reviewers (Mo and Lu) according to the selection criteria. Disagreements were resolved through group discussion. The data collected from each study were as follows: first author’s name, publication year, ethnicity, cancer type, genotyping method, control source, numbers of cases and controls, genotype distribution in cases and controls, and Hardy–Weinberg Equilibrium (HWE). Eligible studies were divided into hospital-based (HB) and population-based (PB) subgroups, according to the control source. Based on the main cancer type of the included studies, cancer types were classified as gastrointestinal cancer (GIC), including oesophageal, gastric, colon, and rectal cancer; gynaecological cancer (GC), including ovarian, cervical, and endometrial carcinoma; liver cancer, lung cancer, breast cancer (BC), and others, including gallbladder carcinoma, pancreatic cancer, nasopharyngeal carcinoma, and lymphoma.

### Statistical analysis

All of the analysis was performed using Stata version 12.0 software (Stata Corp, College Station, TX). The strength of association between the *LAPTM4B* polymorphism and susceptibility to multiple cancers was assessed by odds ratio (OR) and 95% confidence interval (95% CI) in all genetic models (allele model: *2 vs. *1; co-dominant model: *2/2 vs. *1/1, *2/1 vs. *1/1; dominant model: *2/1 + *2/2 vs. *1/1; recessive model: *2/2 vs. *2/1 + *1/1). Subgroup analyses were carried out according to control source, cancer type, tumor grade based on histopathologic differentiation, and tumor stage based on TNM. The stability of the results was assessed using sensitivity analysis by deleting each single study involved in the meta-analysis one at a time to reflect the influence of the individual study to the pooled ORs. Heterogeneity was evaluated by the Chi-square-based Q-statistic and I^2^ statistic. The DerSimonian–Laird random-effects model was used to assess pooled OR when there was a significant difference in terms of heterogeneity (p_Q_ < 0.1 or I^2^ ≥ 50%). Otherwise, the Mantel–Haenszel fixed-effects model was used. Potential publication bias was estimated by funnel plots and Egger’s test. An Egger’s test p value <0.05 was considered statistically significant. The HWE was detected by a goodness-of-fit Chi-square test. All p values were two-sided, and p < 0.05 for any test was considered to be statistically significant. This article was prepared based on the PRISMA guidelines, and the checklist was shown in Additional file 1.

## Results

### Characteristics of eligible studies

**Figure 1:**
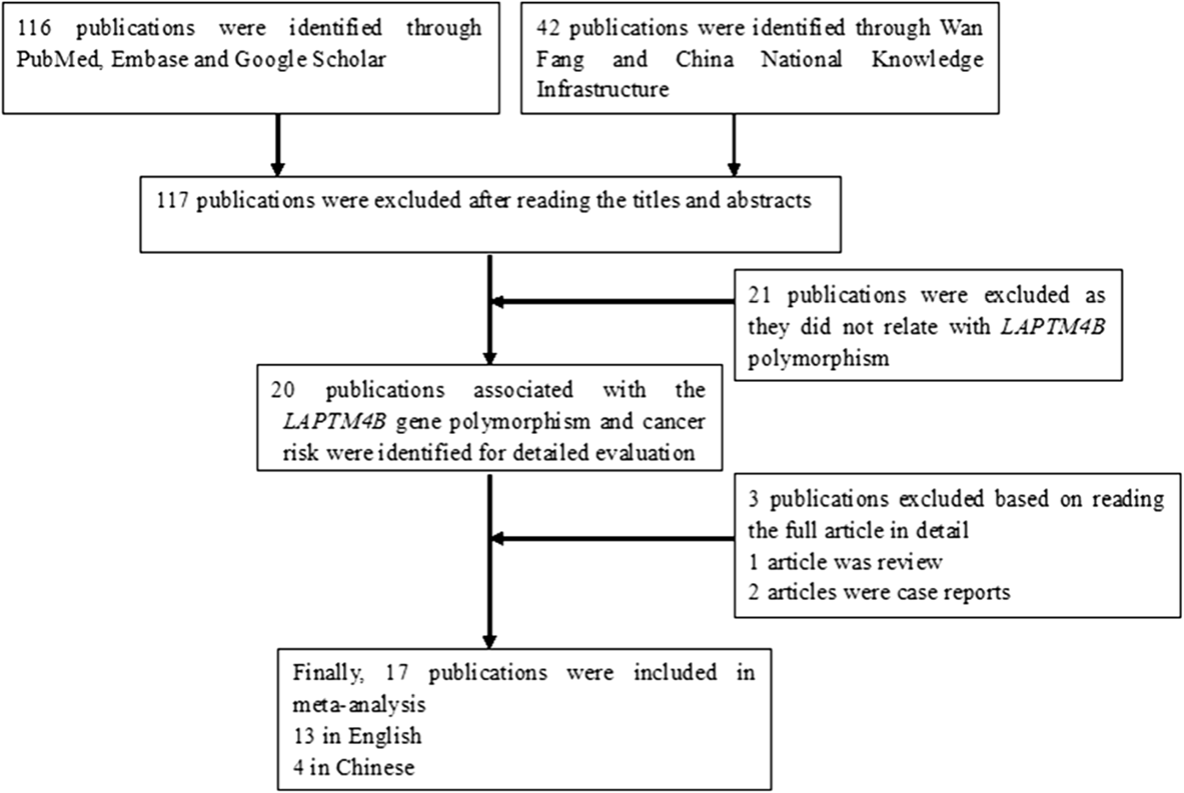
Flow chart illustrates the detailed study selection process of this meta-analysis.

Figure 1 graphically illustrates the detailed study selection process. A total of 20 published studies associated with the *LAPTM4B* gene polymorphism and susceptibility to multiple cancers were identified for detailed evaluation according to our search criteria. After reading the full articles in detail, three studies were excluded because they were case reports [12,13] and a review article [14]. Thus, 17 studies of Chinese populations, totalling 4160 cases and 4148 controls, were included in the meta-analysis [15-31]. Table 1 lists the main characteristics of the included studies. Among these studies, twelve were published in English [17,18,21-29,31] and five were published in Chinese [15,16,19,20,30]. The study of Qi et al. was a postgraduate thesis [16]. The Zhai et al. [17] study only gave the allele frequencies. The study of Cheng et al. [24] included three cancers and shared controls, so the cancer cases were combined into a cancer group in the overall analysis and subgroup analyses stratified by control source and TNM stage, while each cancer was counted as a separate comparison in the subgroup analysis stratified by cancer type. Thirteen of the 17 eligible studies were HB studies and the remaining four were PB studies. The studies focused on the following cancer types: four liver cancer [15-18]; two lung cancer [19,20]; two breast cancer [21,22]; one cervical carcinoma [23]; one colon, rectal, and oesophageal cancers [24]; one gallbladder carcinoma [25]; one gastric cancer [26]; one nasopharyngeal carcinoma [27]; one ovarian cancer [28]; one pancreatic cancer [29]; one lymphoma [30]; and one endometrial carcinoma [31]. All of the patients were confirmed by histopathologic examination. Genotype distributions in the controls of all the studies were in agreement with HWE.

**Table 1:** Characteristics of all the eligible studies

**First author**	**Year**	**Control source**	**Cancer types**	**Genotyping method**	**Case/control**	**HWE**	**Case**			**Control**		
							*1/1	*1/2	*2/2	*1/1	*1/2	*2/2
Sun	2008	PB	Liver cancer	PCR	190/175	0.587	72	110	8	99	67	9
Qi	2010	HB	Liver cancer	PCR	86/78	0.799	27	51	8	36	34	7
Zhai	2012	PB	Hepatocellular carcinoma	PCR	102/135		37	52	13			
Wang	2011	HB	Primary liver cancer	PCR	303/515	0.941	107	156	40	272	205	38
Deng	2005	HB	Lung cancer	PCR	162/134	0.285	54	91	21	67	59	8
Li	2006	PB	Lung cancer	PCR	131/104	0.155	70	56	5	57	36	11
Li	2012	HB	Breast cancer	PCR	208/211	0.185	90	100	18	129	76	6
Fan	2012	HB	Breast cancer	PCR	732/649	0.356	326	342	64	346	262	41
Yang	2012	PB	Gallbladder carcinoma	PCR	91/155	0.851	34	45	12	88	57	10
Wang	2013	HB	Nasopharyngeal carcinoma	PCR	134/327	0.07	74	48	12	163	145	19
Wang	2010	HB	Pancreatic cancer	PCR	58/156	0.977	24	26	8	74	67	15
Xu	2012	HB	Ovarian cancer	PCR	282/365	0.64	122	115	45	231	108	26
Meng	2011	HB	Cervical carcinoma	PCR	317/416	0.835	127	153	37	225	163	28
Meng	2013	HB	Endometrial carcinoma	PCR-RFLP	283/378	0.072	93	135	55	200	140	38
Liu	2007	HB	Gastric cancer	PCR	214/350	0.484	88	107	19	199	133	18
Cheng	2008	HB	Colon/rectal/esophageal cancer	PCR	701/350	0.484	362	293	46	199	133	18
Sun	2007	HB	Lymphoma	PCR	166/350	0.484	72	71	23	199	133	18

*HB* hospital-based, *PB* population-based, *HWE* Hardy-Weinberg Equilibrium, *PCR* polymerase chain reaction, *PCR-RFLP* polymerase chain reaction–restriction fragment length polymorphism.

### Results of meta-analysis

**Figure 2:**
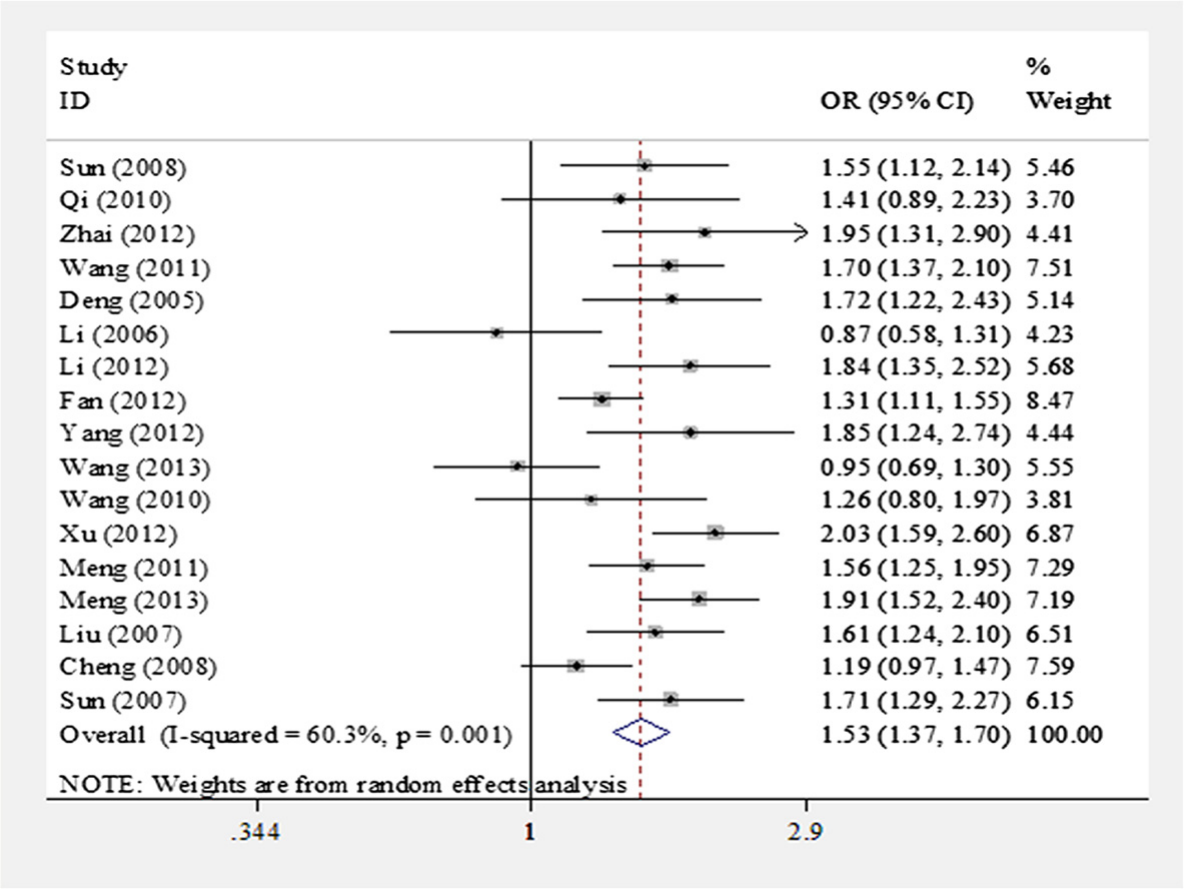
**Forest plots of *LAPTM4B *** polymorphism and overall susceptibility to multiple cancers in allele genetic model (*2 vs. *1) using a random-effect model.

As shown in Table 2, obvious heterogeneity existed among the studies; therefore, the random-effects model was used to assess pooled OR. Overall, the *LAPTM4B* polymorphism was significantly associated with overall susceptibility to multiple cancers in the Chinese population in all genetic models (*2 vs. *1, OR = 1.53, 95% CI = 1.37–1.70, p < 0.001, Figure 2; *2/2 vs. *1/1, OR = 2.18, 95% CI = 1.72–2.75, p < 0.001; *2/1 vs. *1/1, OR = 1.62, 95% CI = 1.41–1.86, p < 0.001; *2/1 + *2/2 vs. *1/1, OR = 1.70, 95% CI = 1.47–1.97, p < 0.001; *2/2 vs. *2/1 + *1/1, OR = 1.76, 95% CI = 1.50–2.05, p < 0.001).

**Table 2:** Meta-analysis of eligible studies included in the study

**Comparison models**	**No. of studies**	**Test of association**			**Mode**	**Test of heterogeneity**			**Publication bias**
		**OR**	**95% CI**	**P value**		***x***^**2**^	**P**_**Q**_**Value**	**I**^**2**^	**P**_**bias**_
*2 vs. *1	17	1.53	1.37-1.70	<0.001	R	40.26	0.001	60.3	0.705
*2/2 vs. *1/1	16	2.18	1.72-2.75	<0.001	R	27.30	0.026	45.0	0.360
*2/1vs. *1/1	16	1.62	1.41-1.86	<0.001	R	31.27	0.008	52.0	0.595
*2/1 + *2/2 vs. *1/1	16	1.70	1.47-1.97	<0.001	R	38.05	0.001	60.6	0.698
*2/2 vs. *2/1 + *1/1	16	1.76	1.50-2.05	<0.001	R	22.05	0.110	32.0	0.265

*OR* odds ratio, *95% CI* 95% confidence interval, *R* random-effects model.

The subgroup analysis results are provided in Table 3. In the subgroup analysis by control source, there was a significant association between the *LAPTM4B* polymorphism and susceptibility to multiple cancers in both the HB (all genetic models) and PB (allele, heterozygote, and dominant comparison models) subgroups. When stratified by cancer type, increased cancer susceptibility was found in all genetic models for liver cancer and GC. There was a significant association with risk of breast cancer in four genetic comparisons, except the homozygous model (*2/2 vs. *1/1, OR = 2.41, 95% CI = 0.97–6.01, p = 0.059). In the lung cancer and GIC subgroups, significant associations were only found in the heterozygote model (lung cancer: *2/1 vs. *1/1, OR = 1.56, 95% CI = 1.11–2.29, p = 0.012; GIC: *2/1 vs. *1/1, OR = 1.33, 95% CI = 1.03–1.73, p = 0.030). Moreover, to investigate the relationship between *LAPTM4B* genotypes and cancer clinical parameters, we analysed the distribution of histopathologic differentiation and classification of TNM and found that the *LAPTM4B* polymorphism was associated with histopathologic differentiation (heterozygote and dominant models). Significant association was found in the allele, heterozygote, and dominant comparison models in the TNM I–II subgroup and in all genetic models in the TNM III–IV subgroup.

**Table 3:** **Stratified analysis of** ***LAPTM4B*** **gene polymorphism on cancer susceptibility**

	**N**	*2 vs. *1			*2/2 vs. *1/1			*2/1 vs. *1/1			*2/1 + *2/2 vs. *1/1			*2/2vs. *2/1 + *1/1		
		**OR (95% CI)**	**p**	**P**_**Q**_	**OR (95% CI)**	**p**	**P**_**Q**_	**OR (95% CI)**	**p**	**P**_**Q**_	**OR (95% CI)**	**p**	**P**_**Q**_	**OR (95% CI)**	**p**	**P**_**Q**_
Control source																
HB	13	1.53 (1.36-1.73)	<0.001	0.002	2.33 (1.96-2.76)	<0.001	0.234	1.58 (1.36-1.84)	<0.001	0.007	1.67(1.52-1.83)	<0.001	0.001	1.86(1.58-2.19)	<0.001	0.612
PB	4	1.49 (1.06-2.092)	0.020	0.023	1.16 (0.35-3.80)	0.811	0.016	1.87 (1.40-2.49)	<0.001	0.247	1.72(1.09-2.72)	0.019	0.073	0.88(0.30-2.54)	0.806	0.029
Cancer type																
Liver cancer	4	1.66 (1.43-1.94)	<0.001	0.714	2.15 (1.42-3.25)	<0.001	0.312	2.03 (1.61-2.57)	<0.001	0.847	2.06 (1.64-2.58)	<0.001	0.963	1.52 (1.02-2.24)	0.037	0.220
Lung cancer	2	1.24 (0.63-2.41)	0.534	0.013	1.13 (0.13-9.50)	0.912	0.003	1.59 (1.11-2.29)	0.012	0.268	1.49 (0.77-2.89)	0.234	0.058	0.90 (0.14-5.90)	0.915	0.007
Breast cancer	2	1.51 (1.09-2.11)	0.014	0.058	2.41 (0.97-6.01)	0.059	0.075	1.49 (1.23-1.81)	<0.001	0.187	1.55 (1.29-1.87)	<0.001	0.101	1.64 (1.13-2.38)	0.009	0.116
GC	3	1.81 (1.58-2.07)	<0.001	0.253	2.90 (2.16-3.90)	<0.001	0.638	1.89 (1.56-2.29)	<0.001	0.584	2.07 (1.73-2.48)	0.007	0.400	2.14 (1.62-2.84)	<0.001	0.716
GIC	4	1.26 (0.97-1.65)	0.084	0.008	1.49 (0.77-2.88)	0.236	0.024	1.33 (1.03-1.73)	0.030	0.084	1.36 (1.00-1.84)	0.050	0.020	1.32 (0.76-2.30)	0.331	0.069
Others	4	1.39 (1.01-1.92)	0.043	0.020	2.32 (1.56-3.46)	<0.001	0.257	1.26 (0.81-1.96)	0.316	0.020	1.39 (0.89-2.19)	0.150	0.011	2.10 (1.43-3.09)	<0.001	0.546
Histopathologic differentiation																
poor	4	1.61 (0.96-2.70)	0.069	0.006	2.34 (0.64-8.59)	0.200	0.008	2.03 (1.10-3.75)	0.024	0.050	2.07 (1.05-4.12)	0.037	0.015	1.56 (0.62-3.95)	0.346	0.060
moderate	4	1.29 (0.96-1.72)	0.091	0.341	0.98 (0.44-2.20)	0.959	0.333	1.83 (1.23-2.74)	0.003	0.527	1.67 (1.13-2.47)	0.010	0.341	0.73 (0.33-1.59)	0.425	0.523
well	4	1.48 (0.88-2.50)	0.143	0.060	2.14 (0.53-8.60)	0.284	0.045	1.60 (1.00-2.55)	0.049	0.172	1.60 (1.03-2.50)	0.037	0.121	1.35 (0.67-2.73)	0.398	0.146
TNM stage																
I-II	8	1.38 (1.04-1.84)	0.028	0.006	1.89 (0.89-4.00)	0.097	0.005	1.43 (1.16-1.77)	0.001	0.415	1.49 (1.22-1.82)	<0.001	0.096	1.61 (0.81-3.18)	0.175	0.008
III-IV	8	1.56 (1.31-1.87)	<0.001	0.032	2.23 (1.67-2.98)	<0.001	0.075	1.79 (1.40-2.28)	<0.001	0.047	1.86 (1.44-2.40)	<0.001	0.017	1.70 (1.29-2.23)	<0.001	0.276

*OR* odds ratio, *95% CI* 95% confidence interval, *P*_*Q*_ p value for heterogeneity, *HB* hospital-based, *PB* population-based, *GC* Gynecological cancer, *GIC* Gastrointestinal cancer, *TNM* Tumor, Node, Metastasis.

### Sensitivity analysis

In order to test whether the modification of the inclusion criteria of the meta-analysis affected the pooled ORs, we performed a sensitivity analysis by deleting each single study involved in the meta-analysis, one at a time. The pooled ORs were not materially altered, suggesting that our results were statistically robust (data not shown).

### Publication bias

**Figure 3:**
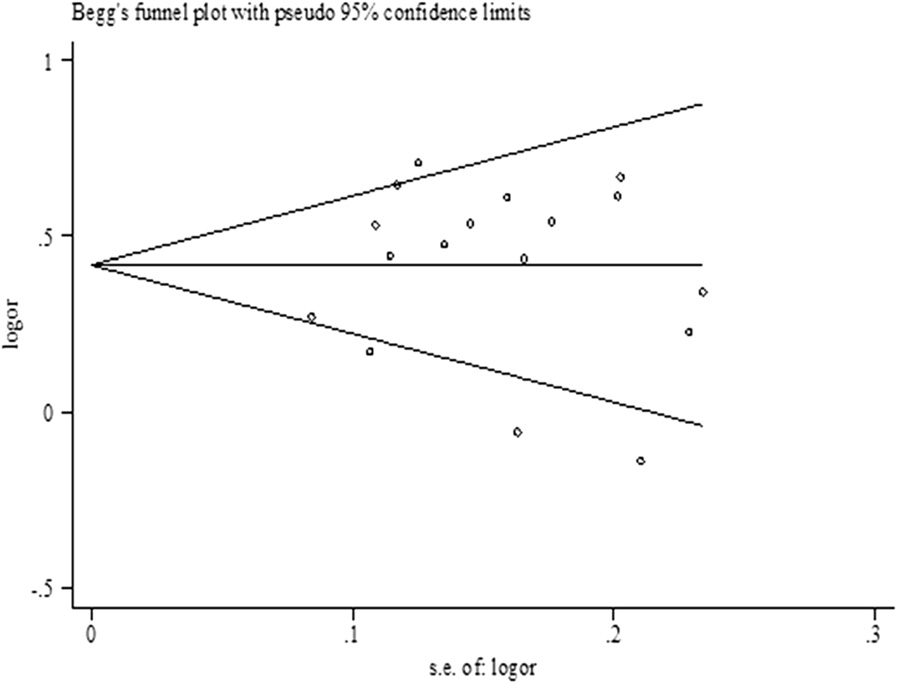
Funnel plot to assess the publication bias of the meta-analysis in allele genetic model (*2 vs. *1).

Egger’s test and Begg’s funnel plot were conducted to estimate the possible publication bias of the studies. The Egger’s test results suggested no publication bias for all the genetic models (Table 2). Figure 3 shows the shape of the Begg’s funnel plots for the allele comparison *2 vs. *1 of the *LAPTM4B* polymorphism in the overall population, indicating no obvious publication bias in the meta-analysis.

## Discussion

Meta-analysis is a valuable tool for estimating the effect of a genetic factor on the risk of a disease when the individual sample sizes are small [32,33]. While previous studies have reported that *LAPTM4B* polymorphism is associated with the risk of several cancers, the results are controversial. Therefore, we carried out this meta-analysis of 17 studies involving 8308 samples, identified through a database search. Overall, we found a significant association between the *LAPTM4B* polymorphism and susceptibility to multiple cancers in all genetic models. This support from our meta-analysis indicates that the *LAPTM4B* polymorphism is a possible susceptibility factor for multiple cancers in the Chinese population, especially for liver cancer and GC.

Cancer is a multifactorial disease and its etiology is still unclear [1]. *LAPTM4B* is a novel cancer-associated gene belonging to the mammalian LAPTM family and upregulated in a wide variety of solid tumors [11,34]. *LAPTM4B* has been shown to promote the transition from G1 to S phase and cell malignant proliferation in *LAPTM4B* cDNA transfected cells by upregulating the expression of cyclin D1 and E [35]. In addition, the overexpression of *LAPTM4B* may activate the integrin-α6 mediated PI3K-AKT signalling pathway and upregulate some proto-oncogenes such as c-myc, c-jun, and c-fos, which regulate apoptosis and cell proliferation [36]. Moreover, knockdown *LAPTM4B* and mutated PPRP motif in the N-terminal region of *LAPTM4B* could attenuate its role in tumorigenesis and metastasis [37]. Previous studies have revealed that the different 19-bp sequence between the *2 and *1 alleles might act as a cis-element, involved in genetic transcriptional regulation. In addition, the extra 53 amino acids at the protein N-terminus produced by LAPTM4B*2 could influence the biological activity and function of tumor cells and then induce oncogenic susceptibility [5].

Previous studies have reported that the increased risk for individuals carrying allele *2 were significantly associated with a susceptibility to liver cancer [17-20], breast cancer [23,24], cervical carcinoma [25], gallbladder carcinoma [27], gastric cancer [28], ovarian cancer [29], lymphoma [32], and endometrial carcinoma [33]. However, no statistical differences were found in nasopharyngeal carcinoma [29] or pancreatic cancer [31]. Deng [21] found that LAPTM4B*2 was a risk factor for lung cancer, while Li [22] reported that LAPTM4B*2 was not closely associated with a susceptibility to lung cancer. Furthermore, the Cheng study indicated that the *LAPTM4B* polymorphism was associated with the development of colon cancer, but not with rectal or oesophageal cancers [26]. Considering these controversial results, we performed the present meta-analysis to establish a more conclusive association between the *LAPTM4B* polymorphism and cancer susceptibility.

To the best of our knowledge, this meta-analysis is the first study to investigate the association between the *LAPTM4B* polymorphism and susceptibility to multiple cancers in a large number of Chinese populations. Our results suggested that the LAPTM4B*2 allele was associated with a risk of susceptibility to multiple cancers, which is consistent with the most of the original research. Significant heterogeneity was observed in all of the genetic model comparisons, which might be due to the control sources and mix of cancers. Most of the studies used hospital-based patients as controls, who were not strictly healthy individuals and could not represent the general population. In this meta-analysis, many cancers were only researched once, and some polymorphisms may play different roles in different cancers. After subgroup analyses were conducted to consider the evidence of heterogeneity, some heterogeneity in the control source and cancer type subgroups was found. Therefore, we speculated that environment components and difference of clinicopathological characteristic might contribute to the heterogeneity. In order to assess the influence of the individual data set to the pooled ORs, a sensitivity analysis was carried out by deleting each single study involved in the meta-analysis each time. The results did not alter, suggesting that our meta-analysis results were stable and credible.

In further subgroup analyses, we found that the *LAPTM4B* polymorphism was associated with HB or PB study design, liver cancer, lung cancer, breast cancer, GC, and GIC. Two studies researched the relationship of the *LAPTM4B* polymorphism with lung and breast cancers, respectively, and they may lack power of statistical analysis. Thirteen studies researched the relationship of *LAPTM4B* polymorphism with cancer histopathologic differentiation, of which four provided gene frequency data. Eight studies researched the association between *LAPTM4B* polymorphism and tumor TNM stage, and only three found that *LAPTM4B* polymorphism was significantly associated with cancer histopathologic differentiation and TNM stage, while the present meta-analysis indicated that *LAPTM4B* polymorphism was associated with histopathologic differentiation and TNM stage. The small sample sizes in the individual original research studies might account for the false positive correlation. Thus, further studies with larger sample sizes and better design are needed.

Our meta-analysis has several advantages. First, this is the first systematic review of the association between *LAPTM4B* polymorphism and susceptibility to multiple cancers, and it is statistically more powerful than any single study. Second, all of the eligible studies researched Chinese populations. The participants have the same genetic background, which can reduce the effects of ethnicity on pooled ORs. Third, the distributions of the genotypes in the controls of the included studies met HWE, which increased the statistical power of the meta-analysis. Lastly, we analysed subgroups stratified by control source, cancer types, histopathologic differentiation, and TNM stage. Furthermore, the sensitivity analysis and publication bias analysis indicated that our meta-analysis results were stable and credible. However, there were still some limitations. First, the meta-analysis was based on unadjusted data; the controls were not strictly matched with the cases in some of the original research, which might have caused some bias. Second, there is notable heterogeneity among studies in the overall analysis and in some subgroup analysis, which could lead to some skewed results. Third, the sample size of some of the subgroups was relatively small. In addition, only one study investigated the association in several human cancers. Given these limitations, the present meta-analysis results should be considered with caution. Further studies with larger sample sizes, more cancer types, and other populations are needed to replicate our results.

## Conclusion

This meta-analysis indicates that the LAPTM4B*2 allele is associated with elevated susceptibility to multiple cancers. In addition, our results indicate a significant association between the *LAPTM4B* polymorphism and cancer susceptibility in the subgroups stratified by control source, cancer type, tumor stage based on TNM, and tumor histopathologic differentiation. All of these results indicate that *LAPTM4B* may be an important biomarker for tumor initiation and development.

## Consent

Written informed consent was obtained from the patient for the publication of this report and any accompanying images.

## Abbreviations

LAPTM4B: Lysosome-associated protein transmembrane-4 beta; HWE: Hardy–Weinberg Equilibrium; HB: Hospital-based; PB: Population-based; GIC: Gastrointestinal cancer; GC: Gynaecological cancer; BC: Breast cancer.

## Competing interests

All authors declare that they have no competing interests.

## Authors’ contributions

CM, YL, and XQ conceived and designed the experiments; SL, YD, and JW performed the experiments; LX, TL, and XQ analyzed the data; YH, QP, and JW contributed reagents/materials/analysis tools; and CM and SL wrote the manuscript. All of the authors read and approved the final manuscript.

## Supplementary Material

Additional file 1 PRISMA Checklist. Click here for file
