# Supplementary material for: LAPTM4B polymorphism increases susceptibility to multiple cancers in Chinese populations: a meta-analysis
Source: BMC Genet. 2014 Apr 18;15:48. doi: 10.1186/1471-2156-15-48 (PMC4022328; doi:10.1186/1471-2156-15-48)
Supplement: Additional file 1 — PRISMA Checklist. [file 1471-2156-15-48-S1.doc]

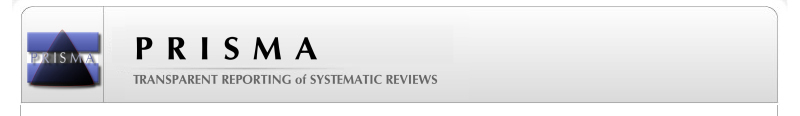
**PRISMA 2009 Flow Diagram**

**Screening**

**Included**

**Eligibility**

**Identification**

Records identified through database searching
(n =158 )

Additional records identified through other sources
(n = 14 )

Records after duplicates removed
(n = 41 )

Records screened
(n = 20 )

Records excluded
(n =21 )

Full-text articles assessed for eligibility
(n = 17 )

Full-text articles excluded, with reasons
(n = 3 )

Studies included in quantitative synthesis (meta-analysis)
(n =17 )
